# Uptake, Translocation, and Fate of Carcinogenic Aristolochic Acid in Typical Vegetables in Soil−Plant Systems

**DOI:** 10.3390/molecules27238271

**Published:** 2022-11-27

**Authors:** Jinghe Zhang, Yinan Wang, Changhong Wang, Kan Li, Weifang Tang, Jing Sun, Xikui Wang

**Affiliations:** 1 Key Laboratory of Fine Chemicals in Universities of Shandong, Jinan Engineering Laboratory for Multi-Scale Functional Materials, School of Chemistry and Chemical Engineering, Qilu University of Technology (Shandong Academy of Sciences), Jinan 250353, China; 2 School of Environmental Science and Engineering, Qilu University of Technology (Shandong Academy of Sciences), Jinan 250353, China; 3 School of Computer Science and Technology, Qilu University of Technology (Shandong Academy of Sciences), Jinan 250353, China

**Keywords:** aristolochic acid, vegetables, uptake, translocation, fate

## Abstract

When Aristolochia plants wilt and decay, aristolochic acids (AAs) are released into the soil, causing soil contamination. It has been demonstrated that aristolochic acid can be accumulated and enriched in crops through plant uptake. However, there is a lack of systematic studies on the migration and accumulation of AAs in a realistic simulated soil environment. In this study, Aristolochia herbal extracts were mixed with soil for growing three typical vegetables: lettuce, celery, and tomato. The contents of AAs in the above-mentioned plants were determined by an established highly sensitive LC-MS/MS method to study the migration and accumulation of AAs. We found that AAs in the soil can be transferred and accumulated in plants. AAs first entered the roots, which were more likely to accumulate AAs, and partially entered the above-ground parts. This further confirms that AAs can enter the food chain through plants and can have serious effects on human health. It was also shown that plants with vigorous growth and a large size absorbed AAs from the soil at a faster rate. The more AAs present in the soil, the more they accumulated in the plant.

## 1. Introduction

**Figure 1:**
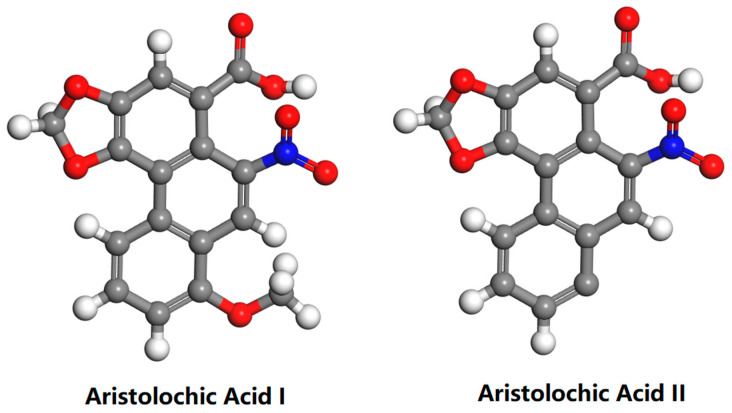
Chemical Structures of Aristolochic Acid I and Aristolochic Acid II.

Aristolochic acids (AAs), highly carcinogenic nitrophenanthrene carboxylic acids, refer to a group of natural products that are widely found in plants of the genus *Aristolochia* [1,2,3]. Various herbs containing aristolochic acid are commonly used in the treatment of various diseases, such as eczema, headache, and stroke [4,5]. AA I and AA II are the major components of AAs (Figure 1) [6]. After ingested AAs enter the human body, AA-induced DNA adducts are generated, which lead to chronic kidney disease in humans and pose a huge threat to human life and health [7,8,9,10]. This kidney disease was first detected in Belgium in 1992, and since then, cases have been detected all over the world, eventually being defined as aristolochic acid nephropathy (AAN) [11,12]. Numerous data have shown that AA-containing plants and their derivatives are nephrotoxic, mutagenic, and carcinogenic to humans [2,13,14]. The International Agency for Research on Cancer (IARC) has classified AAs and the plants that contain them as Group I carcinogens [15].

Plants can absorb and accumulate different environmental pollutants from the soil [16,17,18]. In addition, it has been shown that AAs can be persistent and stable in the environment [19,20]. It is not known whether aristolochic acid, a compound with strong carcinogenicity and nephrotoxicity to humans, will become a new environmental pollutant. Balkan endemic nephropathy (BEN) is a known chronic interstitial nephritis disease in the Balkans of southeastern Europe [11,21]. *A. clematitis L* is widely distributed in cultivated soils in Serbia, and AAs may be the cause of BEN [20,22,23,24]. It has been confirmed that AAs in the environment can be taken up by crops and accumulated through plant root uptake. Chan et al. quantified AAs in maize, wheat grain, and soil samples collected from BEN-occurring villages in Serbia [25]. Pavlovic et al. found that the roots of maize and cucumber could absorb AA I and AA II in nutrient solution [13]. By analyzing agricultural soils and vegetable crops in Serbia, Li et al. determined for the first time that *A. clematitis L* decay released AAs, forming a class of environmental pollutants, and that AAs could enter wheat and maize plants through root uptake [20]. Li et al. observed a more direct effect of AAs on edible plants by watering them with an AA extraction solution. AAs can translocate and bioaccumulate in vegetable crops and are highly persistent in plants [26]. Drăghia et al. found that AAI was present in other plants where *A. clematitis L* grows, and overall, the highest AAI concentrations were found in soil samples, and the lowest ones were detected in cucumber fruits [27]. These studies have shown that *Aristolochia* plants containing AAs undergo wilting and decay and that AAs enter the soil. AAs in the soil are then accumulated via absorption by the roots of other growing crops, which, in turn, are transferred to other parts of the plant. If humans consume plants contaminated with AAs, such as green leafy vegetables and fruits, it will affect their health [28].

Plants containing aristolochic acid are widely distributed [29,30]. However, the route by which AAs enter the food chain has not been fully defined [13]. The contamination of AAs in soil and the potential risk of their uptake and accumulation in vegetables have not yet attracted sufficient attention, and little research has been conducted on the transport of AAs in soil–crop systems. To further determine the absorption of AAs in the soil by edible vegetables and the migration and accumulation rules of AAs in plants, lettuce, celery, and tomato were selected as representatives of edible leaf vegetables, edible stem vegetables, and edible fruits, respectively. They were planted in aristolochic acid-contaminated soil. Through the established high-sensitivity LC-MS/MS method, we detected the contents of AAs in the aforementioned plants and studied the absorption and migration of AAs in plants.

## 2. Results and Discussion

The standard curves for AA I and AA II in lettuce, celery, tomato, and soil were established in the AA standard solution mixture after the above treatments. The concentration of AAs has a good linear relationship with the peak area. The fitted results are shown in Tables S2 and S3, and the correlation coefficients are R^2^ > 0.9990 for lettuce, R^2^ > 0.997 for celery, and R^2^ > 0.9989 for tomato in the concentration range of 50–1000 ng/ mL and R^2^ > 0.9944 for soil in the concentration range of 100–1000 ng/ mL.

The minimum limit of detection (S/N = 3) of AA I and AA II in plants was 1.65 ng/mL and 8.48 ng/mL, respectively. Moreover, the minimum limit of quantification (S/N = 10) of AA I and AA II was 5.5 ng/mL and 35.8 ng/mL, respectively. The relative standard deviation (RSD) of the intra-day precision (*n* = 5) for the three concentrations of AAs was <11.4%, and the RSD of the inter-day precision (*n* = 7) over 2 weeks was <17.4%. The maximum analytical error of AAs was −13.7%. The above accuracy and precision data (Table 1, Tables S4 and S5) indicate that the qualitative and quantitative methods for the determination of AA I and AA II in the plants and soil in this paper are feasible and have good performance.

The results of LC-MS/MS detection and the analysis of AA I and AA II in the plant matrix showed that the three groups of ion transitions (m/z) of AA I peaked at 8.8 min, and the three groups of ion transitions (m/z) of AA II peaked at 8.5 min (Figure S1).

**Figure 2:**
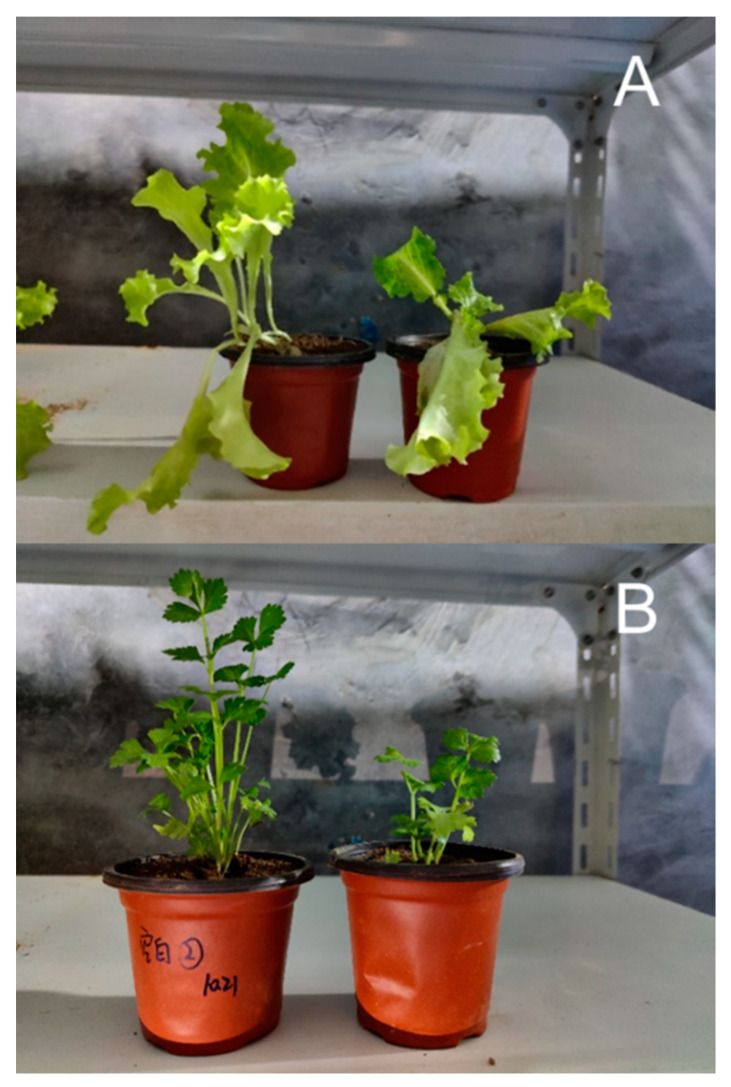
Comparison of the appearance of lettuce (**A**) and celery (**B**) (the left side is the blank plant, and the right side is the plant planted in AA-contaminated soil).

Compared with the lettuce and celery grown in the blank soil, those grown in the soil treated with AAs had shorter plants, easy lodging, few and small leaves, weak growth vigor, underdeveloped root systems, and easy breakage (Figure 2). These results are consistent with the findings of Nikola M. Pavlovic et al., who observed that maize crops grown in areas heavily contaminated with AAs had impaired growth and reduced kernel numbers [13]. AAs are likely to limit the growth of plants and cause adverse effects on their growth and development.

**Figure 3:**
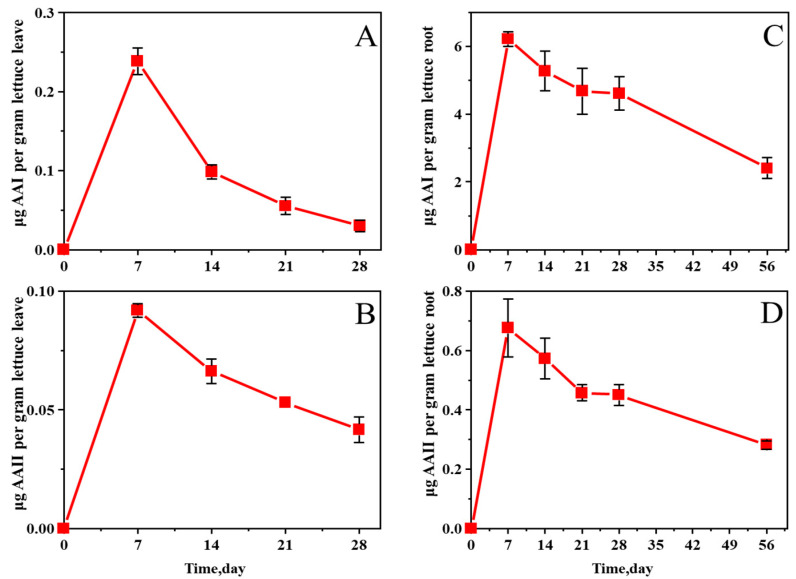
Changes in AA I (**A**) and AA II (**B**) concentrations in lettuce leaves and AA I (**C**) and AA II (**D**) in roots grown in AA-contaminated soil.

On day 7, the absorption of AAs by the leaf samples of lettuce reached the peak; the concentration of AA I reached 0.238 μg/g, and the concentration of AA II reached 0.092 μg/g (Figure 3). This result can be attributed to the fact that the limited amounts of AAs begin to disperse to all parts of the lettuce leaves, thereby leading to a gentle decrease in concentration as the leaves increase in size, especially in large leafy vegetables, such as lettuce. According to the data, the growth cycle of leaf lettuce was approximately 60 days, and the leaves began to wither at about 45 days after the start of the experiment. Thus, 56th-day data were not obtained for the lettuce leaf samples. The trend of AA concentration in the roots was the same as that in the leaves, but the contents of AAs in lettuce roots were much higher than those in lettuce leaves, reaching 6.216 μg/g for AA I and 0.676 μg/g for AA II on day 7 (Figure 3). This phenomenon may be due to the direct contact between roots and soil, where AAs are more directly absorbed into the roots and then translocated to the above-ground parts. The experimental results clearly showed that AAs in soil could be absorbed by lettuce roots and transferred to and accumulated in lettuce leaves.

**Figure 4:**
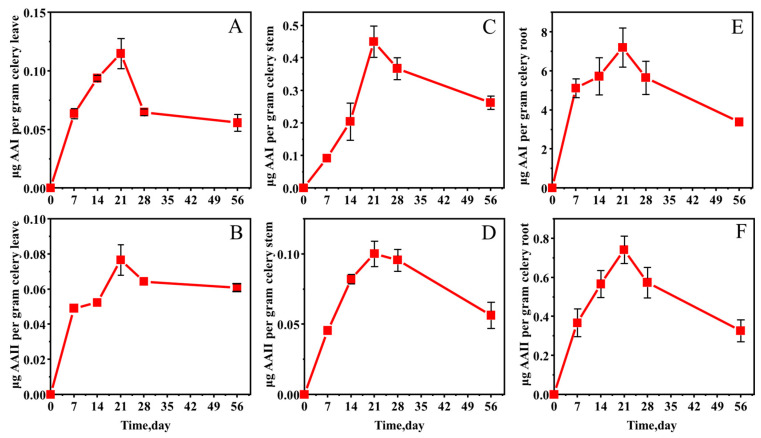
Changes in AA I (**A**) and AA II (**B**) concentrations in celery leaves, AA I (**C**) and AA II (**D**) in stems, and AA I (**E**) and AA II (**F**) in roots grown in AA-contaminated soil.

The concentration of AAs in celery plants gradually increased with time over 21 days after their exposure to AAs (Figure 4). The concentrations of AA I and AA II in celery leaves on day 21 were 0.115 μg/g and 0.077 μg/g, respectively. Subsequently, a decreasing trend was observed. According to previous studies, the leaf clump growth period was approximately 30 to 40 days. In this experiment, the leaf clump had already begun to grow when transplanted. Therefore, the number of leaves was the highest at around 28 days, and the growth rate of the concentration slowed down with the increase in the number of leaves. After 28 days, the number and size of leaves were basically stable, and the concentration changes tended to be gentle.

Consistent with the pattern of changes in leaf concentrations, the concentration of AA I in celery stems reached a peak of 0.450 μg/g on day 21, whereas the concentration of AA II reached 0.100 μg/g. The concentrations of AAs in celery stems began to decrease after 21 days. According to Figure 4, AAs accumulated rapidly in celery stems from 0 to 21 days, with higher concentrations than celery leaves on day 21. In addition, after 28 days, the rate of decrease in the stem was higher than that in the leaves, from which it can be inferred that AAs were transported from the stem to the leaves while accumulating in the stem.

The trend in celery root samples was roughly the same as that in stems and leaves. On day 21, in the roots, the concentration of AA I reached 7.200 μg/g, and AA II reached 0.741 μg/g. The concentrations of AAs in celery roots were much higher than those in leaves and stems. Compared with lettuce roots, the maximum absorption concentration in celery roots was higher but not much different. The reason may be that the root system of celery is more developed. The transfer pattern of AAs in celery further verified that AAs in the soil migrated to the roots first and then transported to the stems and finally to the leaves.

**Figure 5:**
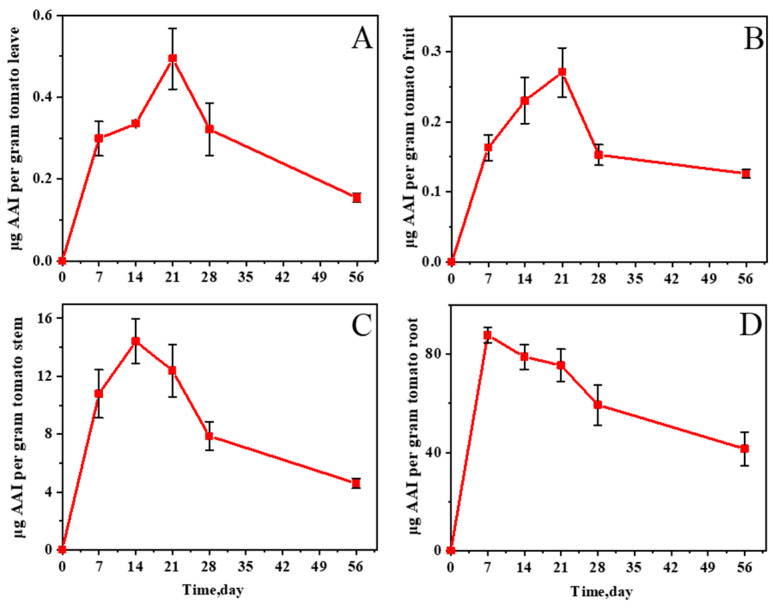
Changes in AA I concentration in tomato leaves (**A**), fruits (**B**), stems (**C**), and roots (**D**) grown in AA-contaminated soil.

In the tomato samples, roots, stems, leaves, and fruits have the highest absorption rate from 0 to 7 days (Figure 5), which is presumed to be in the tomato growing period. The concentrations of AAs in leaves and fruits reached their peaks on the 21st day. In leaves, the concentration of AA I was 0.494 μg/g, and AA II was 0.351 μg/g; in fruits, AA I was 0.270 μg/g, and AA II was 0.268 μg/g. With the ripening of fruit leaves, the concentrations of AAs accumulated in leaves and fruits leveled off from 28 days to 56 days. Figure 5C shows the changes in the AA I content in tomato stems. The concentration of AAs in stems peaked on day 14 at 14.422 μg/g for AA I and 2.914 μg/g for AA II, which were much higher than the concentrations in leaves and fruits. After 14 days, the concentrations of AAs in the stems began to decrease. In the roots, sufficient amounts of AAs had been absorbed after 7 days, and the concentrations were significantly higher compared with those in lettuce and celery, with an AA I concentration of 87.764 μg/g and an AA II concentration of 10.322 μg/g. The patterns of changes in the AA II content in the leaves, fruits, stems, and roots of tomatoes were consistent with the changes in AA I (Figure S2).

By comparing celery and lettuce, we can find that tomatoes absorb the most AAs. It can be presumed that tomatoes require more nutrients when they bear fruit and absorb them faster from the soil, and the plants are larger and require more soil. Therefore, AAs are accumulated more in plants that are actively growing, flowering, and fruiting, and the more AAs that are present in the soil, the more they are accumulated. In addition, a number of factors played a role in the results, such as the daily watering quantity and the nutrient requirements of different plants at different growth stages.

**Figure 6:**
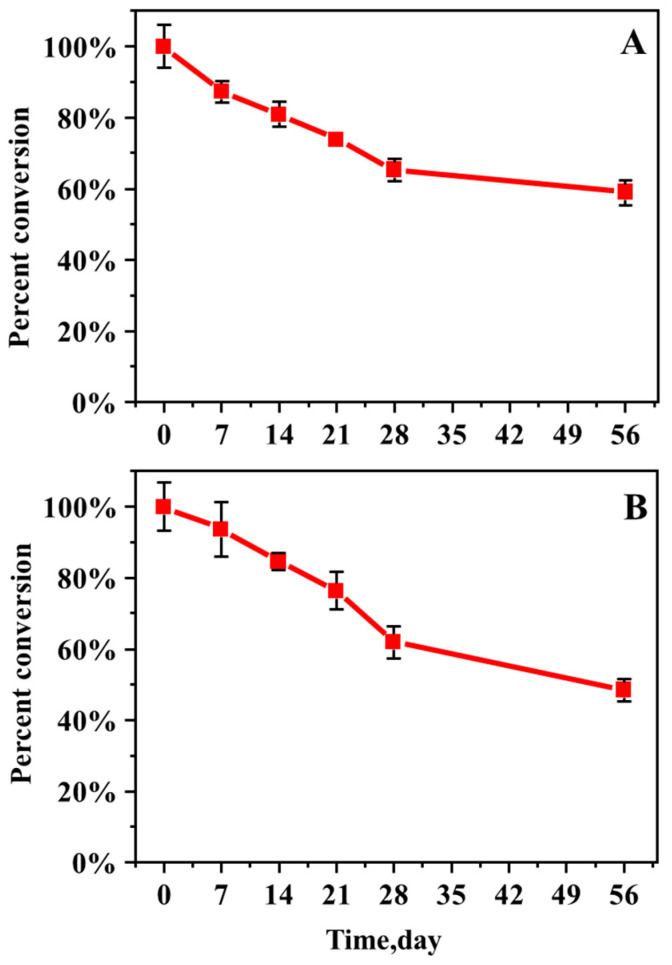
Percentage change in AA I (**A**) and AA II (**B**) from initial soil concentration in the soil where lettuce was grown.

The data showed that the percentage change in AAs from their initial concentrations in the soil where plants were grown decreased slowly (Figure 6, Figures S3 and S4). This result indicates that a portion of AAs in the soil is absorbed by the plant and that AAs are stable in the soil, which is consistent with previous studies [2]. In addition, the concentrations of AAI and AAII in AA-contaminated soil without plant growth also decreased slowly, but at a lower rate than in AA-contaminated soil with plant growth (Figure S5).

## 3. Materials and Methods

### 3.1. Chemicals

AAs are nephrotoxic, mutagenic, and carcinogenic to humans. Thus, they should be treated with caution.All chemicals used were high-performance liquid chromatography grade unless noted otherwise. AA I (90% purity) and AA II (97% purity) were purchased from Sigma–Aldrich (St. Louis, MO, USA). Methanol was supplied by Sinopharm Chemical Reagent Co., Ltd. (Shanghai, China) and Macklin Co., Ltd. (Shanghai, China). Ammonium acetate was purchased from Shanghai Aladdin Co., Ltd. (Shanghai, China). Formic acid was purchased from Tianjin Damao Chemical Reagent Factory (Tianjin, China). Dried *A. debilis fruit* and lettuce, celery, and tomato seedlings were purchased from the Internet. All water in this experiment was ultrapure water.

### 3.2. Preparation of Herbal Extracts

Dried *A. debilis fruit* was crushed and homogenized using a pulverizer. Each gram of powder was extracted with 100 mL of methanol at 50 °C for 60 min through ultrasonication [31]. The extraction solution was filtered with gauze. The extraction process was repeated three times. The final herbal solution was obtained.The herbal solution was poured into the soil at a rate of 2.33 kg/L. Then, it was dried and sieved through a 10-mesh sieve. The soil was mixed and stirred evenly, and the methanol solution in the soil was evaporated to dryness at room temperature. The final soil contaminated with AAs was obtained.

### 3.3. Cultivation of Lettuce, Celery, and Tomato in AA-Contaminated Soil

Lettuce and celery were each planted in 300 g of AA-contaminated soil. After bearing fruits, the tomato was planted in 2 kg of AA-contaminated soil. In addition, blank soil control groups were set up. Samples (n = 5) were collected on days 7, 14, 21, 28, and 56 after AA exposure. Leaf, root, and soil samples were taken from lettuce samples. Leaf, stem, root, and soil samples were taken from celery samples. Similarly, leaf, fruit, stem, root, and soil samples were taken from tomato samples. Then, the samples were stored at −20 °C until sample processing began.

### 3.4. Sample Preparation

Plant samples were washed with ultrapure water, air-dried, and chopped. The sample was weighed to 500 mg, and 1.5 mL of extraction solvent (methanol/water/formic acid, 80:18:2; *v*/*v*/*v*) was added. After 60 min of ultrasonic extraction at 50 °C, the samples were centrifuged at 14,000 rpm for 8 min [26]. The supernatant was filtered through a 0.22 μm filter and centrifuged at 19,000 rpm for 5 min. The samples were dried in a metal bath at 50 °C with a nitrogen blower and reconstituted by adding an equal volume of methanol. A total of 200 μL of the reconstituted samples was transferred to the sample vials with 250 μL lined tubes, and the Shimadzu LCMS-8040 liquid chromatography–mass spectrometer was used for LC-MS/MS detection [32,33]. Soil samples were processed as above.

### 3.5. HPLC-MS-MS Analysis

Sample extracts were analyzed using an HPLC system. The sample extract (10 μL) was injected into a Prevail C18 column (150 × 4.6 mm inner diameter, 5 mm; HiCHROM). Moreover, it was eluted by 20 mM ammonium acetate in water (A) and acetonitrile (B) mobile phases in a gradient in the column at a flow rate of 0.7 mL/min. The solvent gradients were prepared as follows: 0–7 min, 10% to 100% (B, *v*/*v*); 7–10 min, 100% (B, *v*/*v*); 10–15 min, 10% (B, *v*/*v*) [6].

The MS/MS analysis was performed using a SHIMADZU LCMS-8040 Tandem Quadrupole Mass Spectrometer equipped with an ESI source. The analysis was performed in positive ion mode using multiple reaction monitoring (MRM) mode and the following optimized source parameters: atomization gas flow rate: 3.0 L/min; drying gas flow rate: 14.0 L/min; desolvation temperature: 250 °C; heating block temperature: 400 °C; and interface voltage: 4.5 V. The m/z values of the MRM ion pairs for the target analytes are listed in Table S1.

### 3.6. Calibration and Method Validation

AA I and AA II stock solutions were prepared in methanol at a concentration of 20 μg/mL and stored at a low temperature. The stock solution was diluted step by step and mixed with blank lettuce, celery, and tomato extracts at 1:1 to prepare AA I and AA II working standard solutions at concentrations of 50–1000 ng/mL, respectively. After vortex mixing, the standard solutions were analyzed by the LC-MS/MS method. The calibration curves were established by plotting the concentrations of AAs versus the corresponding detected peak areas.

A series of spiked samples at low concentrations was tested according to the pretreatment described above. The lowest concentration of 3 times the signal-to-noise ratio and the lowest concentration of 10 times the signal-to-noise ratio were taken as the limit of detection (LOD) and limit of quantification (LOQ), respectively. Precision was assessed by spiking blank plant and soil extracts with three concentrations of AAs (50, 500, and 1000 ng/g) on the same day (*n* = 5) and on separate days over 2 weeks (*n* = 7) of analysis. The accuracy of the method was assessed by adding AAs (50, 500, and 1000 ng/g) to blank plant and soil samples (*n* = 3) after the pretreatment method to extract AAs and then analyzed by LC-MS/MS.

## 4. Conclusions

In this study, a method was developed for the detection of AAs in plants and soils by ultrasonic high-speed centrifugation pretreatment and analytical detection by the LC-MS/MS method using a liquid-phase tandem triple quadrupole mass spectrometer with an electrospray ionization source. The *Aristolochia* herbal extract was mixed with the soil to grow three typical vegetables, namely, lettuce, celery, and tomato, to observe the accumulation and migration patterns of AAs in plants. The data showed that AAs in soil could be transferred to and accumulated in plants. AAs first entered the roots, which were more likely to accumulate AAs, and partially entered the above-ground parts. This paper reconfirms that AAs can enter the food chain through plants. Moreover, plants that require more nutrients and have larger plants absorb AAs from the soil more rapidly. Therefore, the possibility that the more AAs that are present in the soil, the more they accumulate in the plants cannot be excluded. Given that AAs exist stably in the environment, their entrance into the food chain can have serious effects on human health.

## Figures and Tables

**Table 1:** Limits of detection, intra- and inter-day precision, and accuracy of the developed HPLC-MS/MS method for the determination of AA I and AA II in lettuce.

		Precision		Accuracy		LOD	LOQ
	Concn Added (ng/g)	Intraday *^a^* (%RSD)	Interday *^b^* (%RSD)	Concn Found *^c^* (ng/g)	Error		
AA I	50	8.7%	15.1%	46.7 ± 6.9	−6.7%	2.5	8.5
	500	5.3%	7.8%	473.4 ± 30.0	−5.3%		
	1000	5.0%	8.1%	989.8 ± 33.3	−1.0%		
AA II	50	8.9%	13.4%	47.8 ± 4.3	−4.5%	10.7	35.8
	500	4.6%	13.7%	480.4 ± 57.3	−3.9%		
	1000	9.6%	12.2%	970.9 ± 80.9	−2.9%		

*^a^ n* = 5, *^b^ n* = 7, *^c^ n* = 3.

## Data Availability

The data presented in this study will be available on request.

## Supplementary Materials

The following supporting information can be downloaded at: https://www.mdpi.com/article/10.3390/molecules27238271/s1, Figure S1: Typical LC-MS/MS chromatograms from MRM of AAs in lettuce (A: AA I; B: AA II), celery (C: AA I; D: AA II), and tomato (E: AA I; F: AA II) samples grown in AA-contaminated soil; Figure S2: Concentration changes of AA II in tomato leaves (A), fruits (B), stems (C), and roots (D) grown in AA-contaminated soil; Figure S3: Percentage change of AA I (A) and AA II (B) from initial soil concentration in the soil where celery was grown; Figure S4: Percentage change of AA I (A) and AA II (B) from initial soil concentration in the soil where tomato was grown; Figure S5: Percentage change of AA I (A) and AA II (B) from initial soil concentration in the soil without plant growth; Table S1: MRM transitions of AAs; Table S2: Linear regression parameters of the calibration curves of the developed HPLC-MS/MS method for the determination of AAs in plant; Table S3: Linear regression parameters of the calibration curves of the developed HPLC-MS/MS method for the determination of AA I and AA II in soil; Table S4: Limits of detection, intra- and inter-day precision and accuracy of the developed HPLC-MS/MS method for the determination of AA I and AA II in celery; Table S5: Limits of detection, intra- and inter-day precision and accuracy of the developed HPLC-MS/MS method for the determination of AA I and AA II in tomato.

## Sample Availability

Samples of the compounds are not available from the authors.
