# Supplementary material for: Uptake, Translocation, and Fate of Carcinogenic Aristolochic Acid in Typical Vegetables in Soil−Plant Systems
Source: Molecules. 2022 Nov 27;27(23):8271. doi: 10.3390/molecules27238271 (PMC9739334; doi:10.3390/molecules27238271)
Supplement: Supplementary file 1 [file molecules-27-08271-s001.zip › molecules-2060950-supplementary.pdf]

molecules

MDPI

Supporting Information

Uptake, Translocation, and Fate of Carcinogenic Aristolochic Acid in Typical Vegetables in Soil–Plant Systems

Jinghe Zhang [1,2], Yinan Wang [1,*], Changhong Wang [2], Kan Li [3], Weifang Tang [3], Jing Sun [2] and Xikui Wang [2,*]

[1] Key Laboratory of Fine Chemicals in Universities of Shandong, Jinan Engineering Laboratory for Multi-Scale Functional Materials, School of Chemistry and Chemical Engineering, Qilu University of Technology (Shandong Academy of Sciences), Jinan 250353, China
[2] School of Environmental Science and Engineering, Qilu University of Technology (Shandong Academy of Sciences), Jinan 250353, China
[3] School of Computer Science and Technology, Qilu University of Technology (Shandong Academy of Sciences), Jinan 250353, China
* Correspondence: wangyn@qlu.edu.cn (Y.W.); xikuiwang@126.com or xk_wang@qlu.edu.cn (X.W.)

Table S1. MRM transitions of AAs.

	Quantification ion transition	Collision energy 1 (eV)	Confirmatory ion transition	Collision energy 2 (eV)
**AA I**	359→298	–10	359→296	–10
			359→324	–13
**AA II**	329→268	–7	329→238	–10
			329→294	–20

Table S2. Linear regression parameters of the calibration curves of the developed HPLC-MS/MS method for the determination of AAs in plant.

	Lettuce		Celery		Tomato	
	**AA I**	**AA II**	**AA I**	**AA II**	**AA I**	**AA II**
**Linear range (ng/ mL)**	50–1000	50–1000	50–1000	50–1000	50–1000	50–1000
**Slope**	829.78	158.21	632.53	100.06	464.62	57.529
**Intercept**	2483.9	–2722.1	–9219.4	–1311.5	–11408	–2009.8
**R^2**	0.9990	0.9993	0.9970	0.9987	0.9989	0.9999

Table S3. Linear regression parameters of the calibration curves of the developed HPLC-MS/MS method for the determination of AA I and AA II in soil.

	Lettuce and Celery soil		Tomato soil	
	**AA I**	**AA II**	**AA I**	**AA II**
**Linear range （ng/mL）**	100~1000	100~1000	100~1000	100~1000
**Slope**	1545.6	255.6	773.99	228.49
**Intercept**	–29607	1279.3	55989	–629.47
**R^2**	0.9981	0.9944	0.9981	0.9986

Table S4. Limits of detection, intra- and inter-day precision and accuracy of the developed HPLC-MS/MS method for the determination of AA I and AA II in celery.

		Precision		Accuracy			
	**Concn added (ng/g)**	**Intraday[a] （%RSD）**	**Interday[b] （%RSD）**	**Concn found[c] (ng/g)**	**Error**	**LOD**	**LOQ**
**AA I**	50	1.8%	5.7%	43.2±1.4	–13.7%	2.0	6.8
	500	6.3%	13.3%	521.2±29.2	4.3%		

	1000	4.5%	7.2%	975.0±59.2	–2.5%		
**AA II**	50	4.0%	9.4%	48.5±2.0	–3.1%		
	500	3.9%	17.4%	499.7±20.4	–0.1%	8.5	28.3
	1000	11.4%	15.2%	1026.2±43.1	2.6%		

[a]n=5，[b]n=7，[c]n=3.

Table S5. Limits of detection, intra- and inter-day precision and accuracy of the developed HPLC-MS/MS method for the determination of AA I and AA II in tomato.

		Precision		Accuracy			
	**Concn added (ng/g)**	**Intraday[a] （%RSD）**	**Interday[b] （%RSD）**	**Concn found[c] (μg/g)**	**Error**	**LOD**	**LOQ**
AA I	50	2.8%	12.2%	51.6±0.7	3.2%		
	500	7.1%	12.1%	433.0±28.4	–13.4%	1.7	5.5
	1000	2.6%	3.9%	931.7±45.9	–6.8%		
AA II	50	4.4%	9.8%	52.6±4.4	5.1%		
	500	2.7%	6.8%	475.4±117.2	–4.9%	8.8	24.4
	1000	3.8%	11.5%	979.7±42.6	–2.0%		

[a]n=5，[b]n=7，[c]n=3.

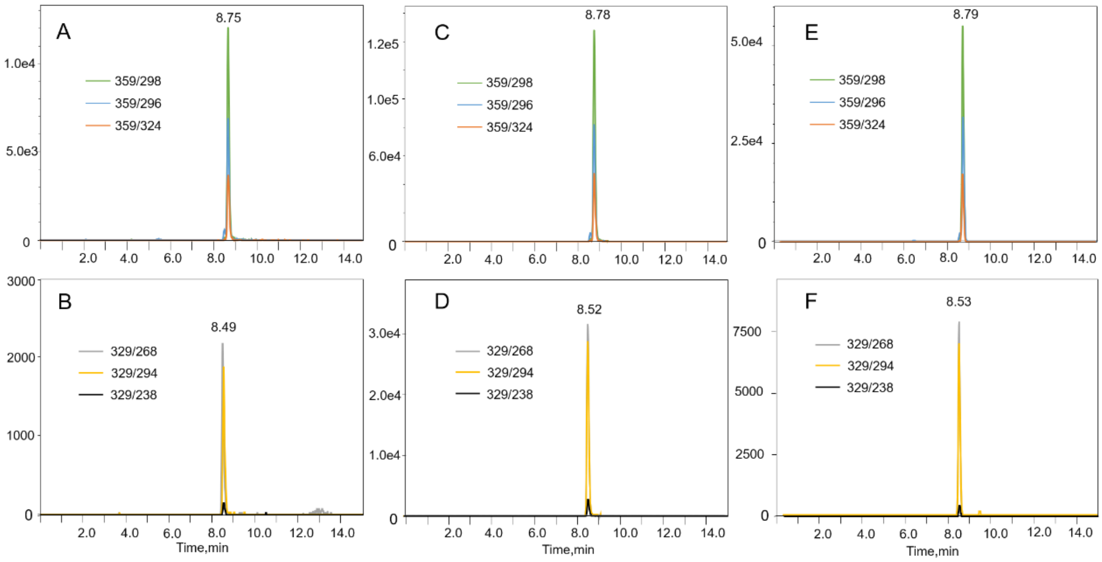

Figure S1. Typical LC-MS/MS chromatograms from MRM of AAs in lettuce (A: AA I; B: AA II), celery (C: AA I; D: AA II), and tomato (E: AA I; F: AA II) samples grown in AA-contaminated soil.

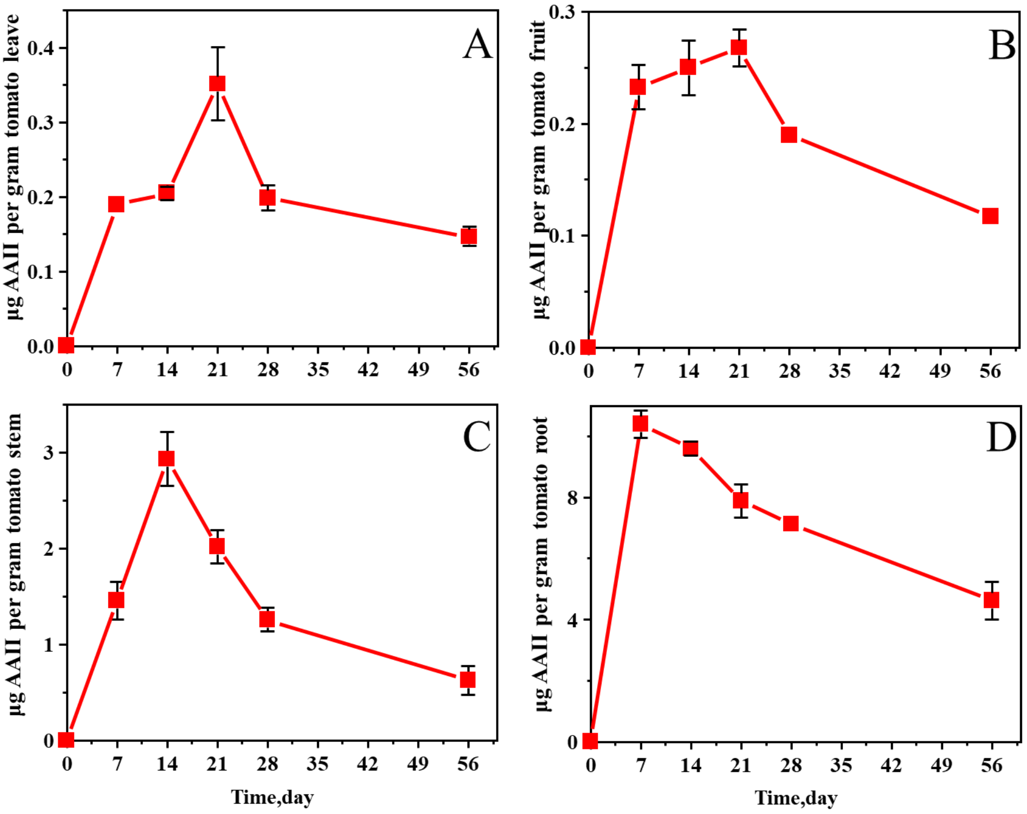

Figure S2. Concentration changes of AA II in tomato leaves (A), fruits (B), stems (C), and roots (D) grown in AA-contaminated soil.

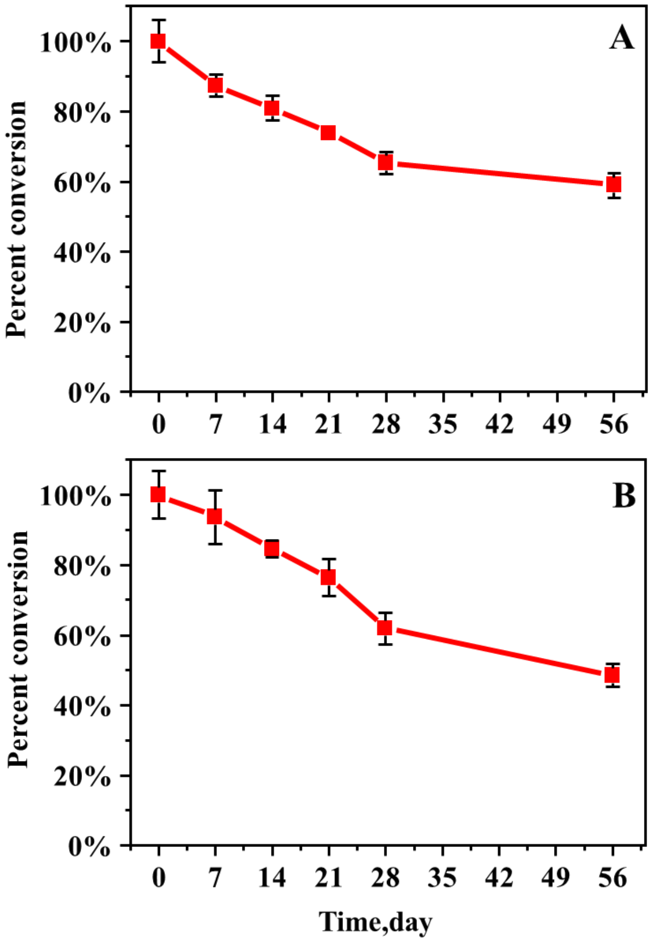

Figure S3. Percentage change of AA I (A) and AA II (B) from initial soil concentration in the soil where celery was grown.

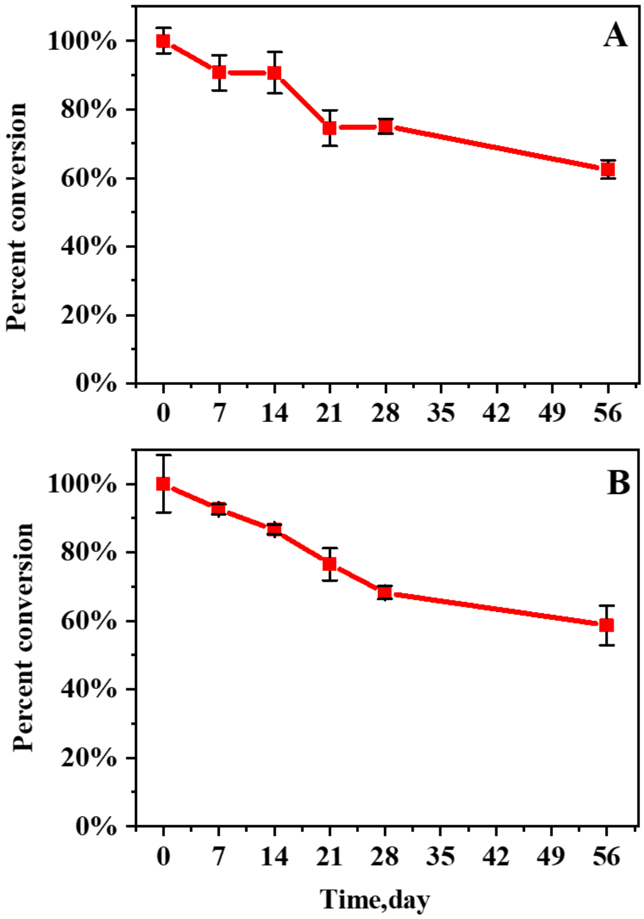

Figure S4. Percentage change of AA I (A) and AA II (B) from initial soil concentration in the soil where tomato was grown.

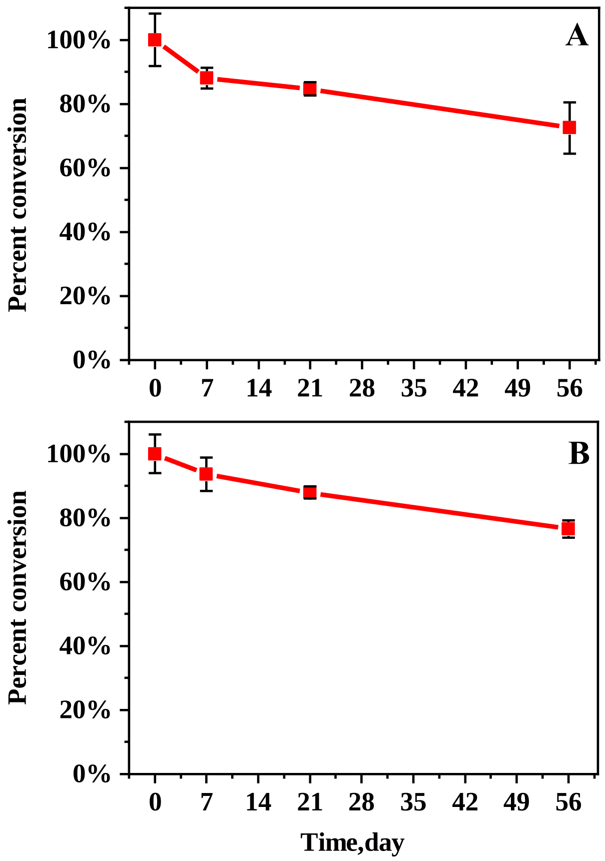

Figure S5. Percentage change of AA I (A) and AA II (B) from initial soil concentration in the soil without plant growth.
